# Quality of reporting in chiropractic mixed methods research: a methodological review protocol

**DOI:** 10.1186/s12998-021-00395-0

**Published:** 2021-09-15

**Authors:** Peter C. Emary, Kent J. Stuber, Lawrence Mbuagbaw, Mark Oremus, Paul S. Nolet, Jennifer V. Nash, Craig A. Bauman, Carla Ciraco, Rachel J. Couban, Jason W. Busse

**Affiliations:** 1 Department of Health Research Methods, Evidence and Impact, McMaster University, Hamilton, ON Canada; 2 Chiropractic Department, D’Youville College, Buffalo, NY USA; 3 Private Practice, Cambridge, ON Canada; 4 Department of Graduate Education and Research, Canadian Memorial Chiropractic College, Toronto, ON Canada; 5 Biostatistics Unit, Father Sean O’Sullivan Research Centre, St. Joseph’s Healthcare-Hamilton, Hamilton, ON Canada; 6 Centre for the Development of Best Practices in Health, Yaundé, Cameroon; 7 Division of Global Health, Stellenbosch University, Stellenbosch, South Africa; 8 School of Public Health and Health Systems, University of Waterloo, Waterloo, ON Canada; 9 Care and Public Health Research Institute, Maastricht University, Maastricht, Netherlands; 10 Department of Anesthesia, McMaster University, Hamilton, ON Canada; 11 Department of Family Medicine, McMaster University, Hamilton, ON Canada; 12 The Centre for Family Medicine Family Health Team, Kitchener, ON Canada; 13 Michael G. DeGroote National Pain Centre, McMaster University, Hamilton, ON Canada; 14 Chronic Pain Centre of Excellence for Canadian Veterans, Hamilton, ON Canada

**Keywords:** Study protocol, Mixed methods research, Reporting quality, Chiropractic, Methodological review

## Abstract

**Background:**

Mixed methods designs are increasingly used in health care research to enrich findings. However, little is known about the frequency of use of this methodology in chiropractic research, or the quality of reporting among chiropractic studies using mixed methods.

**Objective:**

To quantify the use and quality of mixed methods in chiropractic research, and explore the association of study characteristics (e.g., authorship, expertise, journal impact factor, country and year of publication) with reporting quality.

**Methods:**

We will conduct a systematic search of MEDLINE, EMBASE, CINAHL, and the Index to Chiropractic Literature to identify all chiropractic mixed methods studies published from inception of each database to December 31, 2020. Articles reporting the use of both qualitative and quantitative methods, or mixed qualitative methods, will be included. Pairs of reviewers will perform article screening, data extraction, risk of bias with the Mixed Methods Appraisal Tool (MMAT), and appraisal of reporting quality using the Good Reporting of A Mixed Methods Study (GRAMMS) guideline. We will explore the correlation between GRAMMS and MMAT scores, and construct generalized estimating equations to explore factors associated with reporting quality.

**Discussion:**

This will be the first methodological review to examine the reporting quality of published mixed methods studies involving chiropractic research. The results of our review will inform opportunities to improve reporting in chiropractic mixed methods studies. Our results will be disseminated in a peer-reviewed publication and presented publicly at conferences and as part of a doctoral thesis.

**Supplementary Information:**

The online version contains supplementary material available at 10.1186/s12998-021-00395-0.

## Background

Mixed methods designs, which include qualitative and quantitative methods, have been increasingly used in health care research to enrich findings [1, 2]. The explicit mixing or linking of qualitative and quantitative components within a mixed methods study allows researchers to answer questions with a greater breadth and depth of understanding than would be possible with only one methodology alone [1, 2]. This integration of methods, which is central to mixed methods research [1–3], is distinct from “multi-method” research, where investigators use quantitative and qualitative methods in a study without linking or integrating the two components (e.g., adding a series of open-ended questions to the end of a quantitative survey). An integrated mixed methods approach is particularly useful for investigating complex, multilevel programs and interventions [3–6], and is therefore well-suited to address research problems involving knowledge translation, program evaluations, or comparisons of therapeutic interventions within the chiropractic profession. However, little is known about the frequency of use of this methodology in chiropractic research, or the quality of reporting among chiropractic studies using mixed methods.

Researchers conducting mixed methods studies need to make decisions regarding the sequencing or timing of the qualitative and quantitative components (i.e., concurrent or sequential data collection and analysis), as well as the priority or “emphasis” that will be given to each method [1, 2, 7]. For example, Stuber et al. [8] used a sequential, quantitative dominant [7] mixed methods design where supplemental interviews and focus groups (qualitative) were conducted to help explain initial survey results (quantitative) in a study of patient perceptions toward patient-centered care in chiropractic practice. In addition to the mixing or linking of two unique research paradigms—qualitative and quantitative—mixed methods studies may also involve data transformation (i.e., converting qualitative data into quantitative data [‘quantitizing’] or vice versa [‘qualitizing’] in order to further integrate the data [3]). As such, mixed methods studies can become complex investigations that require additional time and resources, and a team of researchers with expertise in quantitative, qualitative, and mixed methodologies [1, 2].

Previous methodological reviews have examined the mixed methods literature [9–14] and highlighted areas for improvement in the quality of reporting. For instance, a review of health services research [9] found that authors of mixed methods studies in health services research typically did not describe or justify the need for a mixed methods design, or integrate data and findings from the individual quantitative and qualitative components. This lack of integration inhibits new insights from being generated within mixed methods studies (i.e., beyond the results obtained from the two separate components), thereby limiting the methodological potential of this research strategy [1–3]. Currently, the quality of reporting in chiropractic mixed methods research is unknown as no methodological reviews have examined this literature.

### Aim

The primary purpose of our methodological review is to examine the quality of reporting and characteristics (e.g., authorship, expertise, journal impact factor, country and year of publication) of chiropractic mixed methods studies. In addition, we will assess the risk of bias of included articles and examine the correlation between reporting quality and risk of bias. We will also use multivariable regression analysis to explore possible factors influencing reporting quality. Our findings will be of interest to educators, researchers, publishers, editors, and consumers of the chiropractic and allied health literature.

## Methods

### Registration

This methodological review was registered with the Open Science Framework (OSF) on December 14, 2020 (https://osf.io/).

### Information sources

**Fig. 1:**
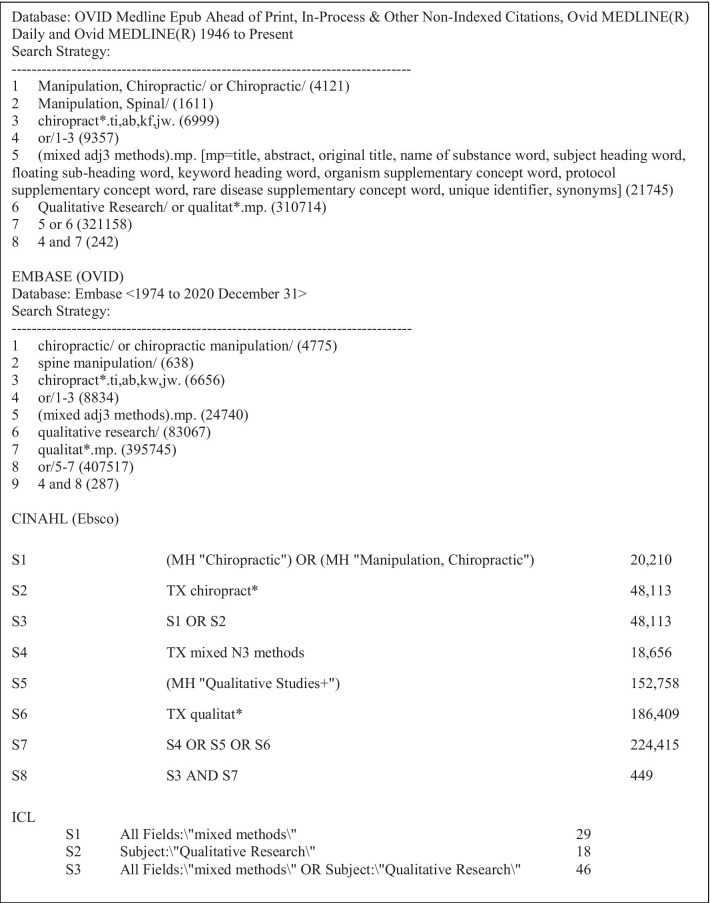
Search strategies for MEDLINE, EMBASE, CINAHL, and the Index to Chiropractic Literature (ICL), from the inception of each database to December 31, 2020

A systematic search of multiple databases including MEDLINE, EMBASE, CINAHL, and the Index to Chiropractic Literature (ICL) will be conducted to identify all published chiropractic mixed methods articles, without time limits to December 31, 2020. Our search strategy was developed by an academic librarian (RJC) (Fig. 1). The reference lists of eligible articles will also be hand-searched, and contact will be made with experts in the chiropractic mixed methods field, to identify additional eligible studies not identified in our electronic database searches. We will update our literature search if more than six months elapses between the time of our database searches and submission of results.

### Eligibility criteria

Articles that meet the following criteria will be included: (1) published in a peer-reviewed journal; (2) written in the English language; (3) authored by one or more chiropractic researchers (i.e., someone with chiropractic credentials or affiliation with a chiropractic educational institution); (4) involved any type of chiropractic intervention (e.g., therapeutic, educational) or non-intervention research (e.g., policy, scope of practice); (5) reported a mixed methods approach (i.e., the use of both qualitative and quantitative methods [1, 2], or mixed qualitative methods [15], in the same research article); and (6) reports primary research. For the analysis, studies that report quantitative and qualitative results in separate papers will be combined and considered as one study. In addition, ‘mixed’ surveys (i.e., those with both closed- and open-ended questions) will only be included when the use of “mixed methods” was explicitly stated in the title or abstract. Protocols, letters, editorials, commentaries, books and book chapters, grey literature (e.g., conference proceedings, abstracts, lectures, dissertations or unpublished manuscripts), and secondary sources of evidence, e.g., clinical practice guidelines or any type of review article will be excluded.

### Study selection

Each title and abstract retrieved from the database searches, as well as those identified through bibliographic-searching and contact with experts, will be screened by pairs of independent reviewers. Online systematic review software (DistillerSR, Evidence Partners, Ottawa, Canada; https://www.evidencepartners.com) will be used to facilitate the literature screening process. We will resolve discrepancies on decisions to include or exclude potentially eligible articles by discussion to achieve consensus or, when not possible, adjudication by a third reviewer. We will also complete full-text screening independently and in duplicate, with discrepancies resolved as previously described. Agreement for full-text screening will be assessed using the kappa (κ) statistic, and the strength of agreement will be interpreted as: poor (κ ≤ 0.2), fair (0.21 ≤ κ ≤ 0.4), moderate (0.41 ≤ κ ≤ 0.6), substantial (0.61 ≤ κ ≤ 0.8), or almost perfect (κ > 0.8) [16].

### Data collection process and assessment of reporting quality

Pairs of reviewers will independently extract data and assess reporting quality of included articles using standardized, pilot-tested data extraction forms. Discrepancies will be resolved by consensus or adjudication by a third reviewer. If a reviewer was an author on an included article, the study will be reviewed by another member of the research team. The following six items from the Good Reporting of A Mixed Methods Study (GRAMMS) criteria [9] will be used to assess reporting quality: (1) describes the justification for using a mixed methods approach to the research question; (2) describes the mixed methods design (i.e., the purpose, priority, and sequence of methods); (3) describes each method in terms of its sampling, data collection, and analysis; (4) describes the integration of the quantitative and qualitative components (i.e., where integration has occurred, how it has occurred, and who among the research team has participated in it); (5) describes any limitation of one method associated with the presence of the other method; and (6) describes any insights gained from mixing or integrating methods. An author checklist of the GRAMMS criteria is provided in Additional file 1. Selected articles will be evaluated on an item-by-item basis, with reviewers rating each item as “yes” (if the item was reported), “yes, but improvements are possible” (if the item was incompletely reported), or “no” (if the item was not reported). For the analysis (see ‘Synthesis of Results’ below), we will sum the scores for each item (1 = “yes”; 0.5 = “yes, but improvements are possible”;  0  = “no”).

The following information will also be extracted from all eligible articles: (1) the first author, (2) year of publication, (3) journal name, (4) number of authors, (5) country where the study was conducted (or, if not available, country of residence of the corresponding author), (6) type of mixed methods design, and (7) whether the list of authors included a mixed methodologist (i.e., graduate-level training or expertise in mixed methods research). We will determine methodological expertise by examining the authors’ published details and affiliations, along with any other information given in the article that explicitly describes an author as having expertise in this area. In addition, the impact factor will be obtained at the year of publication for each journal, either directly from the journal’s website, or from the Journal Citation Report by Thompson Reuters on the Web of Science (https://jcr.clarivate.com/). Journals without an available impact factor will be assigned a value of zero [17].

### Risk of bias of individual studies

We will assess risk of bias of included articles with the Mixed Methods Appraisal Tool (MMAT) (version 2011), which has been validated for systematic reviews of mixed studies (i.e., qualitative, quantitative, and mixed methods studies) [18–20] (Additional file 2). The MMAT is comprised of 11 items in three sections, including: (1) four items for appraising the qualitative component of a mixed methods study or a primary qualitative study; (2) four items for appraising the quantitative component of a mixed methods study or a primary quantitative study (i.e., randomized controlled, non-randomized, or descriptive); and (3) three items for appraising the mixed methods component of a mixed methods study. Pairs of reviewers will appraise each article according to the MMAT, using a similar process as described above.

### Synthesis of results

We will assess all included articles for completeness of reporting on the six items of the GRAMMS instrument (i.e., 0–6 items, where 0 = no reporting of any items and 6 = complete reporting of all six items), and data will be presented as the mean (with standard deviation) or median (with inter-quartile range) number of quality items reported depending on whether the distribution is normal. Risk of bias scores obtained from the MMAT (i.e., 0–11 items) will also be summarized and presented. In addition, we will generate frequencies for individual reporting items on the GRAMMS instrument (i.e., the number of articles reporting a particular item) (Table 1) and describe extracted study characteristics as counts and percentages.

**Table 1:** Reporting quality of included studies according to the Good Reporting of A Mixed Methods Study (GRAMMS) guideline

GRAMMS item	Reporting score and percentage of studies (n = X) reporting each GRAMMS item	
	Score^a^	Percentage
1. Describes the justification for using a mixed methods approach to the research question	X	X
2. Describes the mixed methods design (i.e., the purpose, priority, and sequence of methods)	X	X
3. Describes each method in terms of its sampling, data collection, and analysis	X	X
4. Describes the integration of the quantitative and qualitative components (i.e., where the integration has occurred, how it has occurred, and who among the research team has participated in it)	X	X
5. Describes any limitation of one method associated with the presence of the other method	X	X
6. Describes any insights gained from mixing or integrating methods	X	X

GRAMMS Good Reporting of A Mixed Methods Study

^a^Count scores will be summed as 1 = “yes”; 0.5 = “yes, but improvements are possible”; and  0  = “no”

To examine correlation between the GRAMMS and MMAT instruments, we will compare the item scores for each article between the two instruments (i.e., 0–6 for GRAMMS, 0–11 for MMAT), using Pearson’s *r* or Spearman’s *ρ* for parametric and non-parametric distributions, respectively. Data distributions will be analyzed for normality by visual inspection of histograms, probability plots, and quantile–quantile plots, and then confirmed with the Kolmogorov–Smirnov test. Based on previous findings from research on randomized controlled trials and adherence to CONSORT guidelines [21–23], we predict that chiropractic mixed methods studies with a lower risk of bias (i.e., higher MMAT score) will be correlated with higher reporting quality.

We will use generalized estimating equations (GEEs) to explore the association between reporting quality and article characteristics (i.e., publication date, authorship, and journal impact factor). We will model the dependent variable as the number of GRAMMS items for which complete reporting occurred (maximum value of six) divided by the total number of GRAMMS items (six). The dependent variable will be regressed on the year of article publication (post-2009 versus pre-2009), journal impact factor (higher versus lower), number of co-authors (higher versus lower), and inclusion of an author with training in mixed methods (yes versus no) (Table 2). These factors have previously been shown to be associated with methodological reporting quality [24, 25].

**Table 2:** Unadjusted and adjusted odds ratios for the proportion of Good Reporting of A Mixed Methods Study (GRAMMS) items reported among included studies

Factor	Unadjusted OR (95% CI)	*P*-value	Adjusted OR (95% CI)	*P*-value
Year of publication				
Pre-2009	Reference		Reference	
Post-2009	X	X	X	X
Journal impact factor^a^				
Lower	Reference		Reference	
Higher	X	X	X	X
Number of authors^a^				
Lower	Reference		Reference	
Higher	X	X	X	X
Inclusion of methodologist				
No/unclear	Reference		Reference	
Yes	X	X	X	X

*CI* confidence interval, *OR* odds ratio

^a^This factor will be dichotomized at the median value, calculated across included studies

We will employ a binomial distribution and logit link function in our GEE models to generate crude and adjusted odds ratios, with corresponding 95% confidence intervals and *p*-values. Goodness-of-fit will be assessed by comparing our model’s deviance to its degrees of freedom and by examining the residual plot. We will address over- or under-dispersion by re-running the model with a scale parameter calculated by dividing the deviance by its degrees of freedom. We will also incorporate the journal name as a grouping factor to account for potential similarity or clustering of articles published in the same journal.

We hypothesize that studies published since 2009 (i.e., ≥ 1 year after publication of the GRAMMS criteria [9]), studies published in higher impact journals, those with a greater number of authors, and those that included a mixed methodologist, will be associated with higher reporting quality. A minimum sample of 40 chiropractic mixed methods articles will be needed to guard against over-fitting of our regression model (i.e., minimum of 10 observations per independent variable) [26]. Variance inflation factors (VIFs) will also be explored to assess for multicollinearity among independent variables [27]. If we detect multicollinearity between two or more variables (i.e., VIFs ≥ 10), we will remove the variable(s) that we deem of lower importance [28]. The two-sided statistical significance level ($\alpha$) will be 5%, and all data and comparative analyses will be performed using SPSS v26.0 (IBM SPSS Statistics ©).

### Reporting

Our review will be reported in accordance with an adapted version of the Preferred Reporting Items for Systematic Reviews and Meta-Analyses (PRISMA) guidelines for meta-epidemiological research [29].

### Ethical considerations

This study is a methodological literature review of previously published articles and does not require ethics approval.

## Discussion

This will be the first methodological review to examine the reporting quality of published mixed methods studies involving chiropractic research. The results of this review are important because they will inform areas for improvement regarding reporting of chiropractic mixed methods studies. This may lead to important changes in the quality of evidence generated from these studies, with consequent implications for chiropractic policy, research, editorial, and clinical practice.

Mixed methods research can serve as a powerful tool for investigating complex therapeutic interventions, educational programs or knowledge translation strategies for improving clinical practice [1–6]. However, mixed methods research also requires specialized skills in qualitative and quantitative data integration and analysis [1–3]. Therefore, chiropractors conducting these types of studies should undertake graduate-level training in mixed methods research or, at a minimum, collaborate with researchers possessing mixed methodological expertise.

Previous research has shown that certain authorship factors, such as methodological expertise, as well as having multiple authors on a research project, can significantly improve the reporting quality and conduct of studies [24, 25, 30]. For example, a study on reporting quality among systematic reviews [30] found that including methodologists on research teams was associated with greater concordance with reporting guidelines. Likewise, more recent articles or those published in journals with higher impact factors tend to meet better reporting standards [24, 25]. Yet, no research to date has investigated the influence of these or other factors on the quality of reporting among published chiropractic mixed methods studies.

### Strengths and limitations

Our methodological review has several strengths. First, we will conduct a comprehensive and exhaustive search to identify all eligible studies involving chiropractic mixed methods research. To reduce errors in our methodological procedures, we will perform article screening, data extraction and quality appraisals in duplicate. Moreover, statistical adjustments will be applied at the analysis stage to control for between-group differences when exploring associations, and GEE modelling will be used to account for hierarchical clustering of articles within journals. For our regression model, we have prespecified the anticipated direction of association for each independent variable a priori to give reassurances that associations are unlikely to be spurious if detected. A limitation of this review is we will exclude non-English publications, which may lead to selection bias.

### Knowledge translation

Dissemination of our review will occur via a peer-reviewed publication and a conference presentation. The review findings will also be presented publicly and defended as part of a doctoral thesis.

## Supplementary Information

**Additional file 1**. Good Reporting of A Mixed Methods Study (GRAMMS) checklist. **Additional file 2**. Mixed Methods Appraisal Tool (MMAT), version 2011.

## Data Availability

The datasets to be used and/or analyzed for the current study will be available from the corresponding author on reasonable request.

## Abbreviations

CI: Confidence Interval
CINAHL: Cumulative Index to Nursing and Allied Health Literature
CONSORT: Consolidated Standards of Reporting Trials
EMBASE: Excerpta Medica Database
GEE: Generalized Estimating Equation
GRAMMS: Good Reporting of A Mixed Methods Study
ICL: Index to Chiropractic Literature
MEDLINE: Medical Literature Analysis and Retrieval System Online
MMAT: Mixed Methods Appraisal Tool
OR: Odds Ratio
OSF: Open Science Framework
PRISMA: Preferred Reporting Items for Systematic Reviews and Meta-Analyses
SPSS: Statistical Program for the Social Sciences
SR: Systematic Review
VIF: Variance Inflation Factor
