# Supplementary material for: Quality of reporting in chiropractic mixed methods research: a methodological review protocol
Source: Chiropr Man Therap. 2021 Sep 15;29:35. doi: 10.1186/s12998-021-00395-0 (PMC8442283; doi:10.1186/s12998-021-00395-0)
Supplement: Supplementary file 1 — Additional file 1. Good Reporting of A Mixed Methods Study (GRAMMS) checklist. Additional file 2. Mixed Methods Appraisal Tool (MMAT), version 2011. [file 12998_2021_395_MOESM1_ESM.docx]

**Additional files**

**Additional file 1:** Good Reporting of A Mixed Methods Study (GRAMMS) checklist

**Additional file 2:** Mixed Methods Appraisal Tool (MMAT), version 2011

**Additional file 1** Checklist of items for the Good Reporting of A Mixed Methods Study (GRAMMS) guideline [1,2]

| **Mixed methods reporting** | |
| --- | --- |
| **GRAMMS guideline** | **Location in manuscript where items are reported** |
| 1. Describes the justification for using a mixed methods approach to the research question |  |
| 1. Describes the design in terms of the purpose, priority and sequence of methods |  |
| 1. Describes each method in terms of sampling, data collection and analysis |  |
| 1. Describes the integration of the quantitative and qualitative components |  |
| 1. Describes any limitation of one method associated with the presence of the other method |  |
| 1. Describes any insights gained from mixing or integrating methods |  |

*GRAMMS* Good Reporting of A Mixed Methods Study

**References:**

1. O’Cathain A, Murphy E, Nicholl J. The quality of mixed methods studies in health services research. J Health Serv Res Policy. 2008;13:92-98.
2. Fàbregues S, Hong QN, Escalante-Barrios EL, et al. A methodological review of mixed methods research in palliative and end-of-life care (2014-2019). Int J Environ Res Public Health. 2020;17:3853.

**Additional file 2** Mixed Methods Appraisal Tool (MMAT), version 2011

| **Types of mixed methods study components or primary studies** | **Methodological quality criteria (see tutorial for definitions and examples)** | **Responses** | | | |
| --- | --- | --- | --- | --- | --- |
|  |  | Yes | No | Can’t tell | Comments |
| **Screening questions**  **(for all types)** | - Are there clear qualitative and quantitative research questions (or objectives*), or a clear mixed methods question (or objective*)? |  |  |  |  |
|  | - Do the collected data allow to address the research question (objective)? E.g., consider whether the follow-up period is long enough for the outcome to occur (for longitudinal studies or study components). |  |  |  |  |
|  | *Further appraisal may not be feasible or appropriate when the answer is ‘No’ or ‘Can’t tell’ to one or both screening questions.* | | | | |
| **1. Qualitative** | 1.1. Are the sources of qualitative data (archives, documents, informants, observations) relevant to address the research question (objective)? |  |  |  |  |
|  | 1.2. Is the process for analyzing qualitative data relevant to address the research question (objective)? |  |  |  |  |
|  | 1.3. Is appropriate consideration given to how findings relate to the context, e.g., the setting, in which the data were collected? |  |  |  |  |
|  | 1.4. Is appropriate consideration given to how findings relate to researchers’ influence, e.g., through their interactions with participants? |  |  |  |  |
| **2. Quantitative randomized controlled (trials)** | 2.1. Is there a clear description of the randomization (or an appropriate sequence generation)? |  |  |  |  |
|  | 2.2. Is there a clear description of the allocation concealment (or blinding when applicable)? |  |  |  |  |
|  | 2.3. Are there complete outcome data (80% or above)? |  |  |  |  |
|  | 2.4. Is there low withdrawal/drop-out (below 20%)? |  |  |  |  |
| **3. Quantitative non-randomized** | 3.1. Are participants (organizations) recruited in a way that minimizes selection bias? |  |  |  |  |
|  | 3.2. Are measurements appropriate (clear origin, or validity known, or standard instrument; and absence of contamination between groups when appropriate) regarding the exposure/intervention and outcomes? |  |  |  |  |
|  | 3.3. In the groups being compared (exposed vs. non-exposed; with intervention vs. without; cases vs. controls), are the participants comparable, or do researchers take into account (control for) the difference between these groups? |  |  |  |  |
|  | 3.4. Are there complete outcome data (80% or above), and, when applicable, an acceptable response rate (60% or above), or an acceptable follow-up rate for cohort studies (depending on the duration of follow-up)? |  |  |  |  |
| **4. Quantitative descriptive** | 4.1. Is the sampling strategy relevant to address the quantitative research question (quantitative aspect of the mixed methods question)? |  |  |  |  |
|  | 4.2. Is the sample representative of the population under study? |  |  |  |  |
|  | 4.3. Are measurements appropriate (clear origin, or validity known, or standard instrument)? |  |  |  |  |
|  | 4.4. Is there an acceptable response rate (60% or above)? |  |  |  |  |
| **5. Mixed methods** | 5.1. Is the mixed methods research design relevant to address the qualitative and quantitative research questions (or objectives), or the qualitative and quantitative aspects of the mixed methods question (or objective)? |  |  |  |  |
|  | 5.2. Is the integration of qualitative and quantitative data (or results*) relevant to address the research question (objective)? |  |  |  |  |
|  | 5.3. Is appropriate consideration given to the limitations associated with this integration, e.g., the divergence of qualitative and quantitative data (or results*) in a triangulation design? |  |  |  |  |
|  | *Criteria for the qualitative component (1.1 to 1.4), and appropriate criteria for the quantitative component (2.1 to 2.4, or 3.1 to 3.4, or 4.1 to 4.4), must also be applied.* | | | | |

*These two items are not considered as double-barreled items since in mixed methods research, (1) there may be research questions (quantitative research) or research objectives (qualitative research), and (2) data may be integrated, and/or qualitative findings and quantitative results can be integrated.
